# Prevalence of hearing loss among primary school children in Ethiopia

**DOI:** 10.4314/ahs.v24i4.54

**Published:** 2024-12

**Authors:** Robbert Ensink, Amber Morgan, Alden Smith, Margaretha Casselbrant, Nyasha Makaruse, Glenn Isaacson

**Affiliations:** 1 Department of Oto-rhino-laryngology, Gelre Hospital, The Netherlands; 2 Audiologist, Healing the Children, Greater Philadelphia Area Chapter, Westhampton, NJ, USA; 3 Department of Head & Neck Surgery, UCLA, Los Angeles, CA, USA; 4 Children's Hospital of Pittsburgh of UPMC, Pittsburgh, PA, USA; 5 Departments of Otolaryngology Head & Neck Surgery and Pediatrics, Lewis Katz School of Medicine at Temple University, Philadelphia, PA, USA

**Keywords:** Hearing loss, primary children, Ethiopia

## Abstract

**Objective:**

The objective of this study was to determine the prevalence of hearing loss in children attending primary schools in urban and rural Ethiopia.

**Methods:**

A cross-sectional study was performed to determine the prevalence of hearing loss in children aged 7 to 14 years. A total of 384 children had complete examinations and were included in the study.

**Results:**

The prevalence of hearing loss of all types in the urban school was 6.2% using a cut-off of 40 dB Fletcher index (500–2000 Hz). The prevalence increased to 10.2 % in the same population if a cut-off of 25 dB HL FI was used. In the rural school with a cut-off of 30 dB HL the hearing prevalence was 5.9%. The proportion of conductive hearing loss was lower in the urban school and constituted 16% of all hearing losses. We estimated the proportion of conductive hearing loss in the rural school to be at least 50%. In the urban school the prevalence of sensorineural hearing loss was 5.8% while it was much lower at 1.3% and exclusively unilateral in the rural school. The degree of hearing loss according to WHO criteria was calculated only for the urban population. A prevalence of bilateral severe hearing loss (≥61 dB HL) of 0.5% and of moderate hearing loss (> 41 dB and ≤60dB HL) of 1% was found using WHO criteria.

The prevalence of chronic suppurative otitis media and of dry perforations were similar between schools (2.5 to 2.7%). Otitis media was rare in this study likely due to seasonal influences and exclusion of very young children. These results are compared to similar school studies in Sub-Saharan Africa.

**Conclusions:**

The prevalence of hearing loss in these two Ethiopian cohorts (rural and urban) is in agreement with the data published by WHO for Sub-Saharan Africa. While some of the variation between urban and rural populations may have been real, some of the discrepancy may have resulted from differences in acoustic testing environments. We describe these challenges in hopes of improving universal screening procedures.

## Introduction

Hearing loss in children is recognized as a common disabling condition. The WHO estimates that approximately 5% of the world population, 430 million people have a disabling hearing loss worldwide. About 10% of affected individuals are children less than 15 years of age.^1^ Disabling hearing loss prevalence is highest in sub-Saharan Africa, in South Asia and the Asia-Pacific. In a large questionnaire screening in rural Eastern Ethiopia hearing loss was identified as the most frequent childhood disability accompanied in half by recurrent ear discharge as a result of chronic suppurative otitis media (CSOM).^2^ Different published school studies describe the prevalence of hearing loss ranging between 5 to 10% in the sub-Saharan region.^3^ Variability in cut-off levels, variations in the screening protocols and the hearing frequencies tested likely accounted for some of these diferences. The true prevalence of hearing loss in low-income countries likely exceeds that in high income countries where the prevalence of hearing loss is in the 2 to 4 % range using a cut-off of 25 dB HL. Absence of hearing screening programs at a young age, poor access to hearing care, poverty and malnutrition, lack of appreciation of hearing loss, and parental illiteracy are important contributing factors in the identification of the true prevalence of hearing losses. All lead to lower rates of identification and delayed treatment of hearing loss.

Hearing loss can have many causes. In school children, impacted wax and middle ear effusions are common causes of conductive hearing loss. Impacted wax in the outer ear canal is the most common finding. Wax can cause 40% of failures in school screening for a hearing loss if audiometry is performed without otoscopy.^4^ Conductive hearing loss also may result from tympanic membrane perforation or chronic suppurative otitis media (CSOM). In large school studies performed in Sub-Saharan Africa, prevalence of conductive hearing loss varies between 0.8-21%.^5^ Hearing loss can also present with a normal ear examination, most often representing a sensorineural hearing loss (SNHL). SNHL is often associated with perinatal problems, meningitis, tuberculosis, HIV, ototoxic medications, trauma, or as an inherited trait. Severe bilateral inner ear deafness will interfere with a child's hearing and speech development and may prevent the child from attending school. The prevalence of sensorineural hearing loss varies between 1-2% for the Sub-Saharan continent, but some studies reported far higher prevalences.^1^,^3^,^5^

Today, in most low- and middle-income countries (LMIC's) school screening is not part of integrated school health policies. This is the case in most Sub-Saharan African nations, including Ethiopia. Further descriptive studies are needed to determine the nature and prevalence of the hearing loss in LMICs and to help design optimal screening and public health protocols.

In this article we describe two small school-based studies. One was performed at an urban school in Ethiopia's capital city Addis Ababa, and one at a rural school in the Gurage province. The hearing results from the students in the urban school were previously published as these children served as the control group in a study on HIV related hearing loss.^6^,^7^ That presentation did not include detailed otoscopic findings and prevalence of hearing loss.

We hypothesized that hearing loss and associated otoscopy abnormalities would be more present in the rural school children compared to the urban population. Our aim for both schools, was to calculate a prevalence of hearing loss and report on otoscopic findings. Problems we faced in our hearing screening setting are described. We realized with these confounding factors the true prevalence will vary and differ from the results obtained. We also summarize results of previous school-based studies performed in the sub-Saharan region between 1982 to today.

## Material and methods

### Study populations

The research protocol was reviewed and approved by the Institutional Review Board of the College of Health Sciences of Wolkite University in Ethiopia and the Lewis Katz School of Medicine at Temple University in Philadelphia, USA. The first school, Berhane Zare (BZ) school, is located in a middle class urban setting in the capital city Addis Ababa and was investigated in April 2017. The second school, Sisay and Emetay (SE) school is located in a poor remote rural setting in the Gurage region. At both schools all students aged 7-17 years were informed of the aim of the research, and were screened after written consent from their parent(s) or caretaker was given. On the days of examination, the aim again was explained by the school principal. A total of 179 pupils in BZ school and 238 pupils in SE school had a complete standard ENT examination and pure tone audiometry (PTA) and/ or oto-acoustic emission (OAE) testing.

### Data collection

In selected rooms at their schools all children had ENT examinations by one of the authors (RE, AS, MC, GI) and audiometry by one of the authors (AM, NM) Electricity in the BZ school was available to perform otoscopy and audiometry. At the SE school electricity was absent but mobile audiometry and otoscopy could be performed. Both OAEs and pure tone audiometry were performed for children in grades 1-4 at their schools. For the SE school grades 5-8 underwent pure tone audiometry at a nearby hospital. A standard translated survey for possible causes of hearing impairment and general history was taken from each pupil and if necessary was explained by an Amharic or Afaan Oromo translator. For each child, ear examination was performed with a hand-otoscope prior to audiometry. Wax and purulent discharge were removed from the external ear canal if possible at both schools. Removal of impacted wax was, sometimes, not possible for some of the children in the lowest grades. The appearance of the eardrum was noted as well as the status of the middle ear. After the standard ENT-examination all children of the BZ school were send to a separate room on the top floor of the school for pure tone audiometry (PTA). When ambient noise levels deemed were excessive (e.g., during change of classes) audiometry was temporarily suspended until a quiet environment was restored.

We did not measure the level of back ground noise in the BZ school during the complete testing. So we retrospectively reviewed the audiometry results. In 18 audiograms, noise clearly interfered with hearing screening at 0.5 kHz. These 18 cases were excluded from analysis. Two audiometry technicians performed pure-tone air and bone conduction on all studied children at 0.5-1-2-4-8 kHz. PTA of 0.5-1-2-(Fletcher index) and of 1-2-4 kHz (high Fletcher index) were calculated for both ears; 8 kHz measurements were excluded in the calculations.

At the SE school, examination days were limited because of the first COVID-19 outbreak in March 2020. We therefore chose to examine the youngest children (n=29, in grades 1 and 2) by OAE testing alone. Absent OAEs correspond to a hearing loss greater than 30 dB HL. Thus, our main aim, to establish the prevalence of disabling hearing loss in Ethiopian school children could be realized at both schools. The older children in the SE school were also screened at 30 dB HL for each frequency. All children with absent OAEs had additional pure tone audiometry. All audiometers were calibrated biologically using the hearing levels of the two normal-earing audiologists. At the SE school we excluded 8 children because audiometry or OAE data were missing and 16 children were excluded because of exclusively low-tone hearing loss.

At the SE school, we recorded background noise at the school site continuously by an app (Sound Meter https://play.google.com/store/appsdetails?id=kira.sound). It varied between 33 dB and 85 dB with an average of 51 dBA. When levels were over 50 dB testing was paused. We found similar levels of noise at the hospital testing site.

### Data analysis

Otoscopy results were analyzed descriptively. Given the identified level of background noise, we initially chose to use a cut-off of 40 dB at 0.5-1-2 kHz (Fletcher index) to identify children with hearing loss. We did additional analysis using the high Fletcher index (25 dB hearing level or more at 1-2-4 kHz - excluding 500 Hz) commonly used in other school studies. Hearing losses were categorized into pure conductive, pure sensorineural or mixed hearing losses for the BZ school and categorized as mild (26-40 dB HL); moderate (41-60 dB HL); severe (61-80 dB HL) and profound > 81dB HL according to WHO classification of hearing loss. Since the youngest children at the rural BZ-school underwent only OAE screening, this additional subgrouping according to WHO classification was not possible.

For both schools parental consent and assent forms were collected and kept in a secure location. Paper copies of subject-specific data including ENT history, physical examination findings, and audiograms were collected at the time of the study and identified only by a subject number. De-identified subject data were then transferred to electronic spreadsheet and evaluated using XLSTAT for Microsoft Excel 8

### PuB Med search

In the PubMed search on primary school hearing in Sub-Saharan Africa from 1982 to the present, 24 articles were found. Population-based studies were included only when they were performed in individuals in the age group of the schools we studied and when audiometry was performed. Two articles only reported on audiometry. 17,19 In the remaining 22 articles, audiometry and otoscopy were performed. In 12 articles complete otoscopic and audiometric data were available.^10^,^11^,^13^,^16^,^21^-^27^,^31^ One study was performed in primary school children but their audiometry was used to calculate for a national hearing prevalence for severe and profound hearing loss.^14^ Two studies were not performed in schools, but examined school-aged children.^21^,^25^ A summary of their hearing results; cut-off hearing levels, country and otoscopy results and testing site are summarized in Table 3.

## Results

### Berhane Zare school(urban school)

The examination took place in February 2017 in the dry season. A total of 179 children aged 8 to 17 years were examined. 62% of the subjects were girls. All children were healthy, 1 child had previous surgery for a cleft lip. None of the children had a history of tuberculosis or previous tuberculosis treatment. Four children mentioned a previous head injury. Malaria was not mentioned. 36 children (20.1%) had a history of various otologic complaints. Recurrent episodes of hearing loss were most commonly reported (13/36), followed by periods of ear-discharge (8/36), and otalgia (6/36). Tinnitus and hearing loss related to a previous head injury were mentioned in 4 children.

Otoscopy was not possible due to impacted wax in a total of 29 ears (8.1%); in 15 ears (4%) wax could be removed and otoscopy could be performed. Ten children were excluded from hearing prevalence analysis due to wax. One child had bilateral perforations involving more than 50% of the tympanic membrane and 4 children had unilateral perforations. The total eardrum perforation prevalence in all ears was 1.6 %. With the exclusion criteria as wax; incomplete data, and/or exclusion of children who had audiometry in a noisy environment, 32 children were excluded, leaving a final cohort 147 children. (Table 1) In this group prevalence of otologic complaints was 19% and a total of 6.8% ear drum abnormalities in all ears were present. 2.7% of investigated children had dry eardrum perforations. (Table 2)

**Table 1:** Study enrollment and exclusion criteria by cohort

Urban school	Rural	School
Initial enrollment	179	262
Incomplete examinations	4	8
Noise and impacted wax	28	16
**Final cohort number**	**147**	**238**

**Table 2:** History findings and Otologic examination by cohort

Urban school	Rural	School
**History findings**		
History of otological findings	19%	6.1%
History of ear surgery	0%	0%
**Examination findings**		
Perforation(s)	2.7%	2.5%
Discharge	0%	0%
Other abnormal findings	4.1%	4.7%

Using an outcome parameter of hearing loss (worst ear) >25 dB HL FI, this mild hearing loss was present in 12 % of all children. For all tested children in 2% the hearing loss was conductive. If instead, an endpoint was chosen of PTA >41 dB HL FI (worst ear) and no assessment of background noise was taken, the prevalence of moderate hearing loss was lower at 6.1% and exclusively sensorineural. Of all investigated children in the BZ-school thre were 5 children (3.4%) with a moderate hearing loss (41-60 dB HL) and one child (0.7%) with a bilateral severe hearing loss (61-80 dB HL). Two children had a unilateral severe hearing loss with a normal hearing on the other ear, and two (1.4%) had a moderate bilateral hearing loss.

### Sisay and Emetay school (rural school)

The examination took place in March 2020, in the dry season; just one month before the start of the rainy season. We tested 262 children. A total of 238 children aged 7 to 16 years had a complete ENT and audiometry examination (otoscopy; OAE; PTA or both) There was an equal distribution of girls (49%) and boys (51%). All the children were healthy. One older child with Down syndrome attended grade 2. Forty-nine children (19%) did not return pre-examination health questionnaires given to parents and caretakers. Of the remaining children (n=213) no child reported tuberculosis or previous medication for tuberculosis. In 1 child a meningitis was reported and 20 children reported malaria episodes. One child reported a previous head injury(Tables 1,2).

In 14 children (6.2 %) a history of various otologic complaints was reported. Recurrent episodes of discharge were most commonly reported (9/14), followed by periods of recurrent and fluctuating hearing loss (4/14).

Otoscopy showed wax in 42 children (18%); in 15 children (6%) the wax impaction was bilateral. These children were included in the hearing screening, but excluded when they exclusively had low-frequency hearing loss. OAE testing was not performed in cases of occluded bilateral wax (Table 1,2). A total of 16 children were excluded (Tables 1,2).

In 3 children, unilateral otitis media was present (1.3%). In 5 children (2.1%) a retracted eardrum was present and in one child this was bilateral. Three children had a unilateral foreign body removed. One child had external otitis and one child had an aural polyp suspicious for cholesteatoma. In 6 children (2.5%) a unilateral dry perforation was present.

After exclusion, 238 children had reliable audiological data. In 6 children (2.5%) a conductive hearing loss was found and in 2 of these children the loss was bilateral due to impacted wax or bilateral external otitis. In 3 children (1.2%) a unilateral at least moderate hearing loss of over 41 dB was found. In 5 young children (2.0%) OAEs were absent unilaterally. No bilateral sensorineural hearing loss was found by PTAs and no child had bilateral absent OAEs.

## Discussion

In this study we report on the prevalence of hearing loss and otoscopy findings of school children in Ethiopia and compare this to available data on hearing loss in African school children (Table 3). Only 10 of the 23 articles usedthe current WHO definition of mild hearing loss (<26-40 dB HL). This resulted in decreased 10-13,28 or increased 15-18,31prevalence of hearing loss in some of these studies. When 25 dB HL is set as a cut-off for hearing loss, the prevalence hearing loss in the better hearing ear varies between 0.8-21.3% for Sub-Saharan Africa. Our study could not follow the strict WHO criteria for testing due to noisy school environments during both school testing and logistical problems caused by the COVID outbreak in Ethiopia. These issues led to a shorter protocol of testing and the use of more OAE's for hearing screening than we initially planned. However, OAE testing helped us estimate a rather reliable prevalence rate for mild or greater hearing loss in these rural children, set at 30 dB HL or more. However the limitation in OAE screening was that the protocols to calculate both prevalence of hearing loss differed slightly for both schools.

**Table 3:** Primary school based studies performed in Sub Saharan Africa

Author, country, year	Age, (No. of pupils tested)	Prevalence of mild hearing loss^(#)^	Wax (W), Foreign body (FB)	OME	CSOM	Remarks on otoscopy; audiometry and setting of setting of the screening protocol.
*Bastos^9^*, Angola, 1982.	5-15 yrs. (n=1030)	5.1%	21.5% W	NDA*	3.4%	No significance in hearing between urban and rural populations. All children screened. CSOM prevalence significant doubled in rural areas; 4.6 % versus 2.6 %. No description of study site. Calibration of audiometry on three normal hearing school children.
*Bossoin^15^*, Nigeria, 1986	5-21 yrs (n=2315)	13.5%^#^	35% W; 3,8% FB	NDA	NDA	^#^Definition of unilateral and bilateral hearing loss set at > 20 dB HL. All children screened. No description of study site.
*Prescott^16^*, South Africa, 1991.	6-13 yrs (n=401)	9%^#^	14% W	12%	6% unilateral 15% bilateral	^#^Definition of hearing loss set at >20 dB HL. Otoscopy findings in all children. School screening only in exclusive rural areas. No description of study site.
*Seely^20^*, Sierra Leone, 1992.	5-15 yrs (n=2015)	9.1%	0.8% W	16%	50%	Otoscopy findings in hearing impaired children. 40% of hearing losses bilateral. School screening site in school; and at birth attendant house on peripheral site of village.
*Bastos^21^*, Tanzania, 1993.	6-16 yrs (n=854)	3% (urban and rural children).^#^	10% W	0.5%	1.6%	Otoscopy findings in all children in rural (5 schools) and urban (3 schools) settings. More profound hearing loss in rural settings.^#^ Definition hearing loss set at > 30 dB HL. 20% of hearing loss bilateral. Five healthy normal school pupils at each school site were used for calibration.
*Swart^12^*, Swaziland, 1995.	5-15 yrs (n=2430)	3.3 %^#^	24% W	35%	31%	^#^Otoscopy findings in hearing impaired children. Definition of hearing loss set at > 30-35 dB HL tested at 4 frequencies. None of the 26 schools had sound proofed rooms, Ambient noise measured at 45 dB. Ambient noise levels were confirmed as interfering factor at 0.5 kHz.
*Tshiswaka^17^*, Zaire, 1995.	5-16 yrs (n=2286)	6.9%^#^	NDA	NDA	NDA	Definition of hearing loss set at > 20dB HL. No description of study site.

**Author, country, year**	**Age, (No. of pupils tested)**	**Prevalence of mild hearing loss ^(#)^**	**Wax (W); Foreign body (FB)**	**OME**	**CSOM**	**Remarks on otoscopy; audiometry and setting of setting of the screening protocol.**
*Hatcher^11^*, Kenia, 1995.	5-21 yrs, (n=5368)	5.6%^#^	8.6% W; 0,9% FB.	NDA	1.9% unilateral. 0.6% bilateral.	Otoscopy findings in all children. 40% had bilateral Impairment. ^#^ Definition of hearing loss set at > 30 dB HL. No description of study site. 500 Hz not tested. Ambient noise level tested at 45 dB but not during investigation.
*Von Rooij^22^*, S- Africa, 1995.	<10 yrs (n=2036)	10%	2.5% W	5.2% OME. 0.7% OMA	1.3% wet perf. 0.9% dry perf	Otoscopy in all children. Otitis media with effusion hearing doubled in white children. Definition of loss set at >25-30dB HL. No description of study site.
*Minja^23^*, Tanzania, 1996.	5-20 yrs (n=802)	8.2%	15.7% W	0.4%	2.8%	Otoscopy findings in all children. Higher prevalence of CSOM in rural children (9.44%). Room selected in quiet building.
*Westerberg^13^*, Zimbabwe, 1998	4-20 yrs (n=5528)	2.4%	16% W	23%	20%	Otoscopy findings in hearing impaired children. Definition of hearing loss set at >25-30dB HL. 60% of children with conductive hearing losses. 500 Hz not tested, quite room, Calibration by proven well hearing audiologist. Ambient noise not tested.
Olusanaya^31^, Nigeria, 2000.	4-11 yrs (n=359)	6.4 %	19% W	18.7%	NDA	Otoscopy findings in all hearing impaired children. Better ear calculated. No description of study site. Testing only when ambient noise was < 45 dB.
*North-Mattiassen^19^*, South Africa 2007	6-12 yrs (n=1101)	7.9%	NDA	NDA	NDA	500 Hz was not tested. Only post-screening noise level measurements.
*Clark^36^*, Mozambique, 2010.	1-20 yrs (n=2685)	5%	47% W	27%	NDA	Otoscopy findings only described in the subgroup with mild hearing loss (n=101). Isolated classroom on the campus of a primary school.
*Yamamah^24^*, Egypt, 2010. all losses	7-10 yrs (n=453)	8.5%^#^	9.5% W	10.8%	0.6%	Otoscopy findings in all subjects.^#^ Definition of hearing loss set at >15 dB HL 24% of SNHL. 0.4% moderate to severe hearing loss. Screening tests only carried out when noise levels were below 45dB in quiet section of the schools.

**Author, country, year**	**Age, (No. of pupils tested)**	**Prevalence of mild hearing loss^(#)^**	**Wax (W); Foreign body (FB)**	**OME**	**CSOM**	**Remarks on otoscopy; audiometry and setting of setting of the screening protocol.**
*Taha^18^*, Egypt, 2010.	6-12 yrs (n=555)	20,9%^#^	24.3% W	17.4%	7.0%	Otoscopy findings in students that failed the initial screening tests. ^#^ Definition of hearing loss set at >20 dB HL. Screening in library.
*Adebola^25^*, Nigeria, 2013.	3,5-6 yrs (n=101)	21.3%	21.3%	13.9%	11.9%	Otoscopy findings in pre-intervention group. Screening site in most quiet location, only when noise meter was <45 dB.
*Basanez^10^*, Uganda, 2016.	5-14 yrs (n=639)	3.1%^#^	NDA	12.5%	50%	Otoscopy findings in all hearing impaired_children.^#^ Definition of hearing loss set at > 30 dB HL. 40% conductive losses in 55% SNHL. School screening in library.
*Mahomed-Asmail,^26^* South Africa, 2016.	6-12 yrs (n=1070)	2.2%	6.6% W	7.5%*	NDA	Otoscopy combined with tympanometry findings in all children. Conductive loss 57%. 21% SNHL. More hearing loss in Caucasian children. School screening in classroom, media room and administration.
*Osei^27^* Ghana, 2018.	5-17 yrs (n=210)	21%	22.4% W	NDA	NDA	Definition of hearing loss set at >35 dB HL Only bilateral impacted wax calculated. Appropriate non-specified room in the school.
*Yousuf Hussein^28^* South-Africa, 2019. centers.	3-6 yrs (n=6424)	18.7%	12% W	8.1%	NDA	Excessive and impacted wax and bilateral OME calculated together. In 2/3 of all children a conductive hearing loss Screening in early development childhood
*Solvang^27^* Tanzania, 2019.	6-17 yrs (n=403)	7.1-16,7%	NDA	NDA	NDA	Otoscopy only in hearing impaired children. School screening in a non-specified rooms in three different schools.

**Remarks:**

* NDA: no data available

# different dB hearing level was used to compare the current WHO definition of mild hearing loss.

OME: Otitis Media Effusion; CSOM: chronic suppurative otitis media.

The first published study that investigated hearing in primary school attending children in sub Saharan Africa was performed in The Gambia. No prevalence was given on mild and moderate hearing loss and only prevalence of severe and profound hearing loss was calculated for the whole country and set at 0.3%.^14^ A published study from Nigeria reporting on otoscopy and audiometry reported a high prevalence of otitis media - 40% in the youngest subgroup. Wax was present in 1/3 of all children. The cut-off hearing level was set at 20 dB HL resulting in a prevalence of hearing loss of 13.5%.^15^ A study with similar low cut-off levels reported a 31% prevalence of middle ear anomalies and a 9% rate of hearing loss.^16^ Similar studies in southern African reported 6% prevalence of otitis media with effusion.^12^ A recent study undertaken in 5 schools in South Africa (n=1070) used a cut-off 25 dB HL and yielded a hearing loss prevalence of 2.2%.^27^ Another study in younger South African children in daycare using a similar dB cut-off showed a hearing loss prevalence of 18.7%.^29^ In our Ethiopian cohort the prevalence of hearing loss in the urban school was 6.1% with a cut-off dB hearing level of 40 dB FI. However it was 10.2 % with a dB cut-off hearing level of 25 dB FI. In the rural school when a cut-off of 30 dB HL for audiograms was used (PTA and/or OAE testing), the prevalence was calculated at 5.9% when OAE's were incorporated.

Global acute otitis media(AOM) incidence rate) varies considerably in Sub-Saharan Africa with highest prevalence in the West, Eastern and Central parts of Africa at a rate of 30-35 / 10.000 inhabitants. For Europe these incidence rate is on average 3.64. For both continents 40% occur in children between 0-5 year. 30 In our study we did not find any children with AOM or OME (otitis media with effusion) in the urban and the prevalence in the rural school was low. This discrepancy likely resulted from the exclusion of very young children who have a much high incidence of middle ear disease. It is also possible that seasonal influences in Ethiopia might also have an effect on the prevalence of otitis media since children at both schools were examined before the start of the rainy seasons. In most school studies conductive hearing losses predominate, affecting between 34-60% of students with hearing loss.^10^,^29^

For most African school studies the percentage of pure sensorineural hearing loss varies between 21-24% of all children identified with a hearing loss. In approximately 80% of children with hearing loss the loss is mild to moderate (26-55 dB) and caused by a middle ear problem.^12^,^13^,^28^ Generalized data on the prevalence of OME in Ethiopian children are lacking – as is the case for much of Africa. In the Ethiopian children we studied, the proportion of conductive hearing loss was lower at the urban school and constituted 16% of all the hearing loss. In the remaining 84% of all detected hearing losses, it was sensorineural. In the rural school the percentage of sensorineural hearing loss measured by pure tone audiometry was low, at 1.3% and exclusively unilateral. If we also would have taken the unilateral absent OAEs in 5 children as a sensorineural hearing loss, prevalence in the rural school of sensorineural hearing loss would have been overall 3.2% and still lower than the urban school with a prevalence of 5.8%. However in both schools the number of pupils studied was too low to determine any statistical significance between rural and urban school and the type of hearing loss.

In the largest survey in Africa, from Zimbabwe, 5528 children were studied and a hearing loss prevalence of 2.4 % (dB HL of ≥30 dB) for 1,2,4 kHz was present. Conductive hearing loss made up about 56% of these losses. Among the sensorineural hearing loss reported, in 44% an identified causative factor such as meningitis and/or measles was found.^13^ The second largest group of school children was studied in Kenya.^11^ Wax causing hearing loss was present in 8.6%; in 2.4% of pupils a dry perforation was present and in 1.1% CSOM. Neither the prevalence of OME nor acute otitis media was reported. In 5.6% of all Kenyan children a hearing loss over 30 dB was present. In the majority of children, 81%, the hearing loss was in the range of 30 to 50 dBL. If middle ear problems and wax were excluded prevalence was 3.4%. Profound deafness (>80 dB HL) was present in 0.2% of Kenyan school children although they did not specify if it was unilateral or bilateral. In Uganda, similar prevalence of hearing losses (Table 3) were found with a preponderance of unilateral and/or conductive hearing loss - both more common in girls.^10^ The corrected prevalence was 3.1%. Almost 2% of the total screening population had a sensorineural hearing loss. If the WHO definition of disabling hearing loss in children is taken into account in 1.3% of cases a hearing loss exceeding 31 dB was found consistent with the estimated WHO prevalence of 1.9%.^31^ Some but not all of South African studies described racial and local differences in hearing loss, probably due to more middle ear problems in whites.^16^,^23^ Mohammed-Asmail reported more hearing loss in children of Caucasian descent.^26^ Osei briefly mentions a slight preponderance of hearing loss in males due to higher prevalence to noise exposure.^27^ While our study suggests a higher prevalence of conductive hearing losses in the rural school children, we think significant conclusions cannot be justified by this research mainly because our audiometry testing differed. Similar studies performed between urban and rural schools are few and most studies did not make a comparisons.^22^,^24^ There are several potential confounding factors. The prevalence of CSOM is thought to be higher in rural districts. Illiteracy is more common in rural populations and related to prevalence of hearing loss in children. Severely hearing impaired children will not attend regular school. 1 Also availability and accessibility of medical care in rural areas is poor -largely limited to major cities in many sub-Saharan countries. The recently published study by Birhanu et al in Northern Ethiopia demonstrates how difficult it is to perform audiometry testing in poorly resourced rural settings.^32^ There is no audiology facility located outside Addis Ababa in Ethiopia and 80% of oto-rhino-laryngological care is situated in the capital city.^32^,^33^ We faced similar logical problems in rural and urban settings with COVID-19 as an additional complicating factor in the rural setting hampering us to test all children by PTA as we intended to do. By the replacement with OAE testing we could test all rural children. We could not estimate the level of and characteristics of the hearing loss in the rural children.

Two Northern African studies showed a prevalence of mild hearing loss that varies between 6.7-8.1%.^18^,^24^ A third, school study, without otoscopy, showed a prevalence of 7.7% of pupils who failed both tests. Boys were more likely to have hearing impairments. Only 4% of parents were aware of the presence of the hearing loss in their children.^34^

In all school studies, wax frequently contributes to failure rates of up to 40% of studied children. Bhoola demonstrated in the “middle ear screening protocol” in 1997 in South Africa, that 38% of preschool black and almost half of the preschool Indian children failed the test due to impacted wax.^4^ In Adebola's study, wax was present in 20% of all investigated children.^25^ In our school studies we excluded 24 children (16%) with impacted wax in the urban school and about 18% of all children in the rural school had impacted wax. In a retrospective study of 359 matched children, those children with impacted wax were more likely to have a hearing loss of a permanent nature and they had more reported episodes of otitis media.^35^ These findings suggest that/span>eardrum anomalies are more common in children with impacted wax.

The study performed by Clark in Mozambique was the first to use OAE-screening, in 2685 students ranging from 1 to 20 yrs. Based on OAE screening, 16% of all students failed. PTA testing showed a 5% rate of hearing loss in the same population when >25 dB cut-off was used.^36^ In our rural cohort 30 children had only OAE and 10 children had an abnormal results. We believe OAE screening is a valuable procedure in rural settings, especially when testing time is limited. However selective pure tone screening must supplement OAE screening to establish the type and degree of hearing loss.

Noisy environments are the most significant challenges for successful school hearing screenings. Although we were convinced that a quiet brick classroom would be optimal in the urban school setting, this environment proved unexpectedly noisy and degraded results, especially hearing levels in the low tones. In the end several children were excluded from the analysis at both schools due to background noise. Other primary school screening studies dealt with excessive noise by adopting higher cut-off values at/span> 30 to 40 dB HL and so, finally, did we for both the schools. However in the African school studies (Table 2) only 1 study excluded 0.5 kHz from their prevalence study 13 and 4 did not mention the frequencies tested; 12 studies included 4 kHz, and 3 studies did not include 4 k Hz and only reported the mean Fletcher Index. One study tested the additional 8 kHz test tone.^36^ The American Speech-Language-Hearing-Association (ASHA) advises that school screening should take place in an enclosed, unoccupied and furnished classroom. If these circumstances are met, ambient noise ranges between 30-64 dBA SPL. Our mean ambient noise in the rural school slightly exceeded the upper margins at times. However, during these episodes hearing testing was stopped enabling us almost to not exclude children from the study. Classroom acoustics in developing countries are mostly poor and so more vulnerable to ambient noise because of concrete walls, bare floors and sometimes absent roofs. In warm environments doors and windows often must be left open, admitting more noise.

Classroom noise by itself is concentrated at lower frequencies. For the most part, conventional ear phones used in hearing screening in schools in LMIC countries do not eliminate low frequency ambient noise.^37^,^38^ We encountered similar problems in our schools and stopped testing when noise levels were too high. Table 3 briefly summarizes the site of testing, the calibration procedures and ambient noise testing performed during various studies. Variation in frequencies tested and the lack of a standardized protocol for screening has resulted in considerable study-to-study variation in the estimated prevalence of school hearing loss in the sub-Saharan Africa. We do realize that all these factors are, in our opinion unavoidable for sub-Saharan African school testing. So before setting up a school based screening protocol one should be aware that many factors contribute to variations in hearing prevalence partly due to the hearing frequencies tested and to variations in the testing site. Awareness of these factors needs to be described in detail before hearing screening is initiated.

## Conclusion

In this study audiometry and otoscopy results are presented from two school screenings, one in an urban and one in a rural environment in Ethiopia. We were successful in determining the prevalence of handicapping hearing losses in these children thanks, in part, to the inclusion of selective OAE screening. Our results are comparable to previous studies performed in other the sub-Saharan countries. The proportion of sensorineural hearing losses varied between the rural and urban schools. The prevalence of CSOM and dry perforations was similar between schools. Description of a practical universal screening protocol, based on available resources and accounting for environmental limitations, would be a good first step in the establishment of uniform, nation-wide school screening for Sub- Saharan countries.
